# Genetic susceptibility, screen-based sedentary activities and incidence of coronary heart disease

**DOI:** 10.1186/s12916-022-02380-7

**Published:** 2022-05-24

**Authors:** Youngwon Kim, Shiu Lun Au Yeung, Stephen J. Sharp, Mengyao Wang, Haeyoon Jang, Shan Luo, Soren Brage, Katrien Wijndaele

**Affiliations:** 1 School of Public Health, The University of Hong Kong Li Ka Shing Faculty of Medicine, Pokfulam, Hong Kong SAR China; 2 MRC Epidemiology Unit, University of Cambridge School of Clinical Medicine, Box 285 Institute of Metabolic Science, Cambridge Biomedical Campus, Cambridge, Cambridgeshire CB2 0QQ UK

**Keywords:** Genetic risk, Coronary heart disease, TV viewing, Computer use, Polygenic risk scores, UK Biobank

## Abstract

**Background:**

Whether the associations of time spent in screen-based sedentary activities with CHD vary by genetic susceptibility is currently unknown. The objective of this study was to examine the interplay of genetic susceptibility to CHD and two prevalent types of screen-based sedentary activities (television [TV] viewing and computer use) for CHD incidence.

**Methods:**

This prospective cohort study included 373,026 individuals of European ancestry without prevalent CHD/stroke from UK Biobank data. Genetic susceptibility to CHD was assessed using weighted polygenic risk scores, calculated by summing the number of risk-increasing alleles among 300 single-nucleotide polymorphisms, multiplied by their corresponding effect estimates. TV viewing and computer use were assessed through touch-screen questionnaires. CHD incidence (*n*=9185) was adjudicated over a median 12.6-year follow-up.

**Results:**

Compared with ≥4h/day of TV viewing, the hazard ratio of CHD was 0.84 (95% confidence interval [CI] 0.79–0.90) and 0.94 (0.90–0.99) for ≤1h/day and 2–3h/day of TV viewing, respectively, after adjusting for confounders including the genetic risk. CHD hazards were higher for medium and high genetic risk than for low genetic risk. Across all levels of genetic risk including high-genetic risk, ≤1h/day of TV viewing had lower CHD hazards, compared with ≥4h/day: no evidence of interaction between genetic risk and TV viewing (*p* value: 0.362). Estimates of the population attributable fraction (PAF) suggested that 10.9% (95% CI 6.1–15.3%) of CHD could be prevented if TV viewing time were reduced to ≤1h/day, assuming causality. The PAF values were relatively larger for medium-to-high genetic risk than for low genetic risk, although the CIs were wide and overlapping. No associations were observed for computer use.

**Conclusions:**

Less TV viewing time was associated with lower CHD risk independently of genetic risk. Clinical trials targeted at individuals with high genetic susceptibility should consider reducing TV viewing as as a behavioural target for prevention of an early onset of cardiovascular events.

**Supplementary Information:**

The online version contains supplementary material available at 10.1186/s12916-022-02380-7.

## Background

Coronary heart disease (CHD) presents a substantial clinical and public health burden, with over 9.1 million deaths and 182 million disability-adjusted life years globally [1]. Prevention of CHD is multi-faceted, as CHD is caused by a combination of both genetic and non-genetic environmental traits [2, 3]. Over recent years, the genetic aetiology of CHD has been investigated through Genome-Wide Association Study (GWAS) research [4] identifying a multitude of single-nucleotide polymorphisms (SNPs) associated with CHD risk [5]. Common genetic variants identified from GWAS allow the construction of polygenic risk scores for CHD, making it possible to estimate an individual’s genetic susceptibility to CHD [5]. In addition to the genetic contribution, CHD is also characterised by non-genetic environmental factors including lifestyle behaviours. Of various behavioural traits, sedentary behaviour has recently emerged as an independent modifiable marker of CHD [6]. Sedentary behaviour is defined as any waking behaviour performed in the sitting, reclining or lying posture producing an energy expenditure of ≤1.5METs, but the operationalised definition of sedentary behaviour includes screen-based sedentary activities [7].

Contemporary individuals spend nearly two thirds of their leisure time engaging in screen-based sedentary activities, the most prevalent of which include television (TV) viewing and computer use [8]. Reducing time spent in these screen-based sedentary activities in addition to overall sitting is, therefore, of clinical and public health relevance [9]. Previous research has reported on varying levels of CHD risk according to specific types of screen-based sedentary activities, with more consistent associations for TV viewing than for computer use [10–14]. However, no previous research [10–14] on the role of different types of screen-based sedentary activities in CHD has considered the influence of individuals’ unique genetic susceptibility to CHD. Evidence, however, indicates that up to 40% of CVD risk is attributable to genetic predispositions to CHD [4]. As such, it is critical to explore to what extent the risk of CHD associated with high genetic risk of CHD can be modified by time spent in specific types of screen-based sedentary activities. While few investigations have explored the association of composite healthy lifestyle scores with cardiovascular events in the context of genetics [15–18], no research has examined the underlying interplay of time spent in any screen-based sedentary activities and genetic susceptibility to CHD relative to CHD incidence. Currently, little is known about whether the benefits of less time spent in specific screen-based sedentary activities for CHD risk differ by genetic susceptibility. Whether TV viewing or computer use may play a moderating role in the associations of genetic risk with CHD incidence remains unanswered. Therefore, the purpose of this study is to examine whether the associations of TV viewing and computer use with incident CHD vary by genetic susceptibility to CHD.

## Methods

### Study design and participants

We used data from a large-scale prospective cohort study, UK Biobank, which includes over half a million UK adults 40–69 years of age at recruitment [19]. The key eligibility criteria in the UK Biobank study included living in a place <25 miles away from one of 22 assessment centres in the UK and is registered within the National Health Service database. Between 2006 and 2010, the baseline measurement was carried out collecting information on a wide variety of variables including genotype data, demographic indicators, body composition and lifestyle behavioural outcomes. The present analysis was based on 373,026 participants who were considered white British (based on self-reported ethnicity and principal component analysis of genotype data), had no prevalence of CHD/stroke (based on self-report and hospital admission records) and had no missing data for any covariates (Additional file 1: Fig. S1). The UK Biobank study protocol was approved by the Northwest Multi-Centre Research Ethics Committee (11/NW/0382). Before participation, participants provided signed informed written consent.

### Exposures

#### Polygenic risk scores for CHD

In UK Biobank, the participants were genotyped using the UK Biobank Axiom Array and UK BiLEVE Axiom Array, which were then imputed to a combined haplotype reference panel of the Haplotype Reference Consortium and UK10K [20]. For the current study, we followed an established methodology [21] to calculate weighted polygenic risk scores representing each individual’s genetic susceptibility to CHD. Detailed descriptions about the estimation procedure are provided elsewhere [21]. Briefly, we included 300 uncorrelated SNPs from 240 loci [4] associated with the risk of CHD (Additional file 1: Table S1), which is a combined set of genome-wide significant SNPs and uncorrelated SNPs at a false discovery rate of 5% (the latter of which were identified from a meta-analysis of interim UK Biobank genotype data with the CARDIoGRAMplusC4D 1000 Genomes–imputed GWAS or the MIGen/CARDIoGRAM Exome chip studies) [21]. The weighted polygenic risk score was calculated by summing the number of risk alleles, multiplied by the corresponding effect estimates [4, 21], using PLINK2.0. The calculated continuous polygenic risk score showed a normal distribution (Additional file 1: Fig S2) and was classified into low, medium and high genetic risk according to the tertiles.

#### Two types of screen-based sedentary activities

Each participant in UK Biobank was asked to fill out a touch-screen questionnaire set which included questions asking about various behaviour variables including TV viewing and leisure-time computer use. Time spent on TV viewing and leisure-time computer use (both of which are non-occupational) on a typical day was each reported in 1-h increments. Three categories of TV viewing and computer use were generated: ≤1h/day, 2–3h/day and ≥4h/day; this categorisation strategy has been used in previous research [22, 23] and used herein due to the hourly discrete nature of the reported variables and non-linear trend of associations (Additional file 1: Fig. S3).

### Incidence of CHD

Measured data of participants in UK Biobank were linked with their national death registry and hospital admission records. CHD incidence in this study was ascertained according to a series of algorithms based on both death registry and hospital admission data [24]. Codes of International Classification of Diseases (ICD) were used to adjudicate CHD cases (ICD-9: 410-411,412.X, ICD-10: I21-I24, I25.2) accrued until October 31, 2021, for individuals in England and Wales and November 12, 2021, for individuals in Scotland. Incident CHD was defined as the first observation of CHD events that occurred over a 12.6-year median follow-up (interquartile range: 12.0–13.4years), resulting in a total of 9185 incident CHD cases.

### Confounders

The following variables that may confound the associations between genetic risk, TV viewing/computer use and CHD were included as confounders [25]: age (underlying timescale), sex, body mass index (BMI) (i.e. higher BMI associated with higher sedentary time including TV viewing [26, 27] and higher CHD risk [28], but not acting as a mediator [29]), smoking status (never, previous, current), employment (unemployed, employed), Townsend Deprivation Index (a numerical deprivation score generated based on employment, car ownership, home ownership and household overcrowding according to postcode of participants’ home address), alcohol consumption (never, previous, currently <3times/week, currently ≥3times/week), salt-adding behaviour (never/rarely, sometimes, usually, always), oily fish consumption (never, <once/week, once/week, >once/week), coffee intake (cups/day), fruit and vegetable intake (a composite score generated based on intake of fresh/dried fruit and intake of raw/cooked vegetable ranging from 0 to 4), processed/red meat intake (days/week), hypertension medication use, cholesterol-lowering medication use, glucose-lowering medication use, sleep (≤5, 6, 7, 8, ≥9hours/day) and moderate-to-vigorous physical activity (minutes/day; calculated by summing up moderate activity [frequency×duration] and vigorous activity time [frequency×duration] (multiplied by 2) [30] performed in a typical week; questions derived from the modified International Physical Activity Questionnaire-Short Form). The genotyping array type (UK Biobank Axiom Array, UK BiLEVE Axiom Array) and the first ten principal components of ancestry (to control for population stratification [31]) were adjusted for in models for the polygenic risk score.

### Statistical analyses

Cox regression models (with age as the underlying timescale) using either TV viewing or computer use as the main exposure were established by adjusting for no confounders (Model 1), and confounders (Model 2) with an additional adjustment for the polygenic risk score (as a continuous variable) and mutual adjustment of TV viewing and computer use (Model 3), after excluding the first 2 years of follow-up. Models using the polygenic risk score as the main exposure were also fit with adjustment for sex, the genotyping array type and the first ten principal components of ancestry. Multiplicative interactions between three categories of TV viewing or computer use and polygenic risk scores were tested in models adjusted for confounders. Models were also fit to estimate the joint associations of TV viewing or computer use and genetic risk with incident CHD, with low genetic risk combined with ≤1h of TV viewing or computer use as the reference group; these models did not include the polygenic risk score variable as a potential confounder. The cumulative hazards of CHD across three categories of TV viewing, computer use and polygenic risk score were plotted. Population attributable fractions (PAFs) were calculated to estimate proportional risk reductions in CHD that would occur if ≥2h/day of TV viewing and computer use were reduced to ≤1h/day of TV viewing and computer use, respectively, assuming causality [10, 32, 33]. The calculation of confidence intervals for the PAFs assumed asymptotic normality of the estimates (‘*punafcc*’ command in Stata/MP Version 16.0) [34, 35]. All models were fit using age as the underlying timescale and adjusting for the 2nd-degree genetic relatedness (kinship coefficients between 0.0442 and 0.0884) [36] using cluster-robust standard errors [20]. Log-log plots supported the proportional hazards assumption for each exposure. Nine sensitivity analyses were performed: (1) excluding an additional 2 years of follow-up (4 years in total) to address reverse causality, (2) excluding individuals with poor self-reported health status (i.e. based on the 4-level self-reported health ratings; poor [excluded], fair, good, excellent) to address reverse causality, (3) excluding individuals with the 2nd-degree genetic relatedness, (4) using a weighted polygenic risk score calculated based only on 46 lead SNPs (from 46 loci) which were genome-wide significant at a p value of 5×10^-8^ and in low linkage disequilibrium defined according to *r*^2^<0.001 (Additional file 1: Table S1 and Fig. S4), 5) using values imputed for the covariates missing (using multiple imputation with changed equations), assuming data missing at random, (6) including prevalence of type 2 diabetes and renal dysfunction as confounders, (7) excluding body mass index as a confounder, (8) with CHD follow-up censored on January 1, 2020, to account for potential CHD cases not captured due to participants’ fear of visiting clinics during the COVID-19 pandemic, and (9) with adjustment for education, household income and occupation as individual-level indicators of socio-economic status as opposed to area-level socio-economic status, Townsend Deprivation Index. All data files are available from the UK Biobank database (https://www.ukbiobank.ac.uk/). Statistical analyses were performed using Stata/MP Version 16.0 (StataCorp LP, College Station, TX).

## Results

Characteristics of participants across three categories of TV viewing and computer use are provided in Table 1. On average, individuals spent 2.8 h/day of TV viewing (standard deviation: 1.6) and 1 h/day of computer use (standard deviation: 1.3). Table 2 shows associations of TV viewing and computer use with incident CHD. Compared with ≥4h/day of TV viewing, ≤1h/day and 2–3h/day of TV viewing were associated with 16% (95%CI 10–21%) and 6% (95%CI 1–10%) lower hazards of CHD, respectively, after adjusting for potential confounders including genetic risk for CHD (Model 3). There was no evidence of associations between computer use and CHD incidence (Model 3). The results of the sensitivity analyses were similar to these findings (Additional file 1: Tables S2-S10).

**Table 1:** Characteristics of individuals overall and within three categories of TV viewing and computer use

Variables	All	TV viewing			Computer use		
		≤1h/day	2–3h/day	≥4h/day	≤1h/day	2–3h/day	≥4h/day
Age, years	56.6 (8.0)	54.5 (8.0)	56.2 (8.0)	58.9 (7.4)	56.7 (8.0)	57.1 (8.0)	54.8 (8.1)
Sex, *n* (%)							
Men	169,944 (45.0)	33,316 (44.4)	87,103 (45.5)	47,525 (44.6)	121,287 (41.4)	35,008 (57.8)	11,649 (60.5)
Women	205,082 (55.0)	41,645 (55.6)	104,452 (54.5)	58,985 (55.4)	171,890 (58.6)	25,588 (42.2)	7604 (39.5)
Body mass index, kg/m^2^	27.3(4.7)	25.8 (4.2)	27.2 (4.5)	28.5 (5.0)	27.1 (4.6)	28.0 (4.9)	28.3 (5.1)
Smoking status, %							
Never	207,454 (55.6)	45,862 (61.2)	108,976 (56.8)	52,616 (49.4)	166,034 (56.6)	31,513 (52.0)	9907 (51.4)
Previous	129,536 (34.7)	23,178 (30.9)	65,618 (34.3)	40,740 (38.3)	99,253 (33.9)	23,323 (38.5)	6960 (36.2)
Current	36,036 (9.7)	5,921 (7.9)	16,961 (8.9)	13,154 (12.4)	27,890 (9.5)	5,760 (9.5)	2386 (12.4)
Employment, %							
Unemployed	218,047 (58.4)	54,041 (72.1)	121,299 (63.3)	42,707 (40.1)	173,776 (59.3)	31,407 (51.8)	12,864 (66.8)
Employed	154,979 (41.6)	20,920 (27.9)	70,256 (36.7)	63,803 (59.9)	119,401 (40.7)	29,189 (48.2)	6389 (33.2)
Townsend Deprivation Index	−1.64 (2.9)	−1.69 (2.8)	−1.83 (2.8)	−1.26 (3.1)	−1.68 (2.9)	−1.55 (2.9)	−1.33 (3.1)
Alcohol consumption, %							
Never	11,000 (3.0)	2,252 (3.0)	5,064 (2.6)	3,684 (3.5)	8,939 (3.1)	1,551 (2.6)	510 (2.6)
Previous	11,745 (3.1)	2,263 (3.0)	5,168 (2.7)	4,314 (4.0)	8,893 (3.0)	2,109 (3.5)	743 (3.9)
(<3times/week)	178,219 (47.8)	31,970 (42.7)	90,484 (47.3)	55,765 (52.4)	142,312 (48.5)	27,201 (44.9)	8706 (45.2)
Current (≥3times/week)	172,062 (46.1)	38,476 (51.3)	90,839 (47.4)	42,747 (40.1)	133,033 (45.4)	29,735 (49.0)	9294 (48.3)
Salt-adding behaviour							
Never/rarely	212,554 (57.0)	46,690 (62.3)	110,834 (57.9)	55,030 (51.6)	167,637 (57.2)	34,086 (56.2)	10,831 (56.3)
Sometimes	103,119 (27.6)	19,310 (25.8)	53,217 (27.8)	30,592 (28.7)	81,102 (27.7)	16,732 (27.6)	5285 (27.4)
Usually	41,833 (11.2)	6,979 (9.3)	20,652 (10.8)	14,202 (13.4)	32,341 (11.0)	7,255 (12.0)	2273 (11.6)
Always	15,520 (4.2)	1,982 (2.6)	6,852 (3.5)	6,686 (6.3)	12,097 (4.1)	2,523 (4.2)	900 (4.7)
Oily fish consumption							
Never	39,408 (10.6)	6,815 (9.1)	18,952 (9.9)	13,641 (12.8)	30,745 (10.5)	6,395 (10.6)	2268 (11.8)
<Once/week	126,007 (33.7)	24,611 (32.8)	64,393 (33.6)	37,003 (34.7)	98,465 (33.6)	20,803 (34.3)	6739 (35.0)
Once/week	142,894 (38.3)	29,542 (39.4)	74,586 (38.9)	38,766 (36.4)	113,082 (38.6)	22,888 (37.8)	6924 (36.0)
>Once/week	64,717 (17.4)	13,993 (18.7)	33,624 (17.6)	17,100 (16.1)	50,885 (17.3)	10,510 (17.3)	3322 (17.2)
Coffee intake (cups/day)	2.07 (2.1)	2.05 (2.0)	2.08 (2.0)	2.09 (2.2)	2.03 (2.0)	2.22 (2.1)	2.33 (2.4)
Fruit and vegetable intake	1.59 (1.2)	1.72 (1.2)	1.60 (1.2)	1.47 (1.1)	1.59 (1.2)	1.57 (1.1)	1.54 (1.2)
Red meat intake, days/week (average)	0.90 (0.5)	0.82 (0.5)	0.90 (0.5)	0.98 (0.6)	0.89 (0.5)	0.95 (0.6)	0.94 (0.6)
Hypertension medication use, %	18.6%	11.9%	17.4%	25.6%	18.2%	20.8%	18.6%
Cholesterol-lowering medication use, %	14.6%	8.7%	13.3%	21.2%	14.0%	17.3%	15.7%
Glucose-lowering medication use	0.9%	0.6%	0.7%	1.2%	0.8%	1.1%	1.3%
Sleep							
≤5 h/day	18,047 (4.8)	2,864 (3.8)	8,482 (4.4)	6,701 (6.3)	13,794 (4.7)	3,093 (5.1)	1205 (6.3)
6 h/day	69,250 (18.6)	13,633 (18.2)	35,346 (18.5)	20,271 (19.0)	53,309 (18.1)	12,009 (19.8)	4077 (21.2)
7 /day	148,422 (39.8)	33,455 (44.6)	78,927 (41.2)	36,040 (33.8)	117,415 (39.9)	23,728 (39.2)	7557 (39.2)
8 h/day	109,990 (29.5)	21,044 (28.1)	56,668 (29.6)	32,278 (30.3)	87,800 (29.9)	17,215 (28.4)	5184 (26.9)
≥9 h/day	27,317 (7.3)	3965 (5.3)	12,132 (6.3)	11,220 (10.6)	21,629 (7.4)	4551 (7.5)	1230 (6.4)
Moderate-to-vigorous physical activity (min/day)	56.8 (71.0)	58.0 (69.4)	58.0 (71.7)	53.6 (70.6)	58.5 (72.4)	52.4 (66.8)	43.6 (57.7)
Polygenic risk score for CHD	17.3 (0.6)	17.3 (0.6)	17.3 (0.6)	17.3 (0.6)	17.3 (0.6)	17.3 (0.6)	17.3 (0.6)

Values are means (standard deviations) or percentages, unless otherwise indicated

**Table 2:** Associations of TV viewing and computer use with incident coronary heart disease (CHD).

Comparisons	Number of participants	Number of cases	Crude incident rate per 100,000-person years	Hazard ratio (95% confidence interval)		
	373,026	9185	195.3	Model 1^a^	Model 2^b^	Model 3^c^
**Categories of TV viewing**						
≥4h/day (reference)	106,510	3501	261.8	1.00 (reference)	1.00 (reference)	1.00 (reference)
2–3h/day	191,555	4413	182.5	0.81 (0.78–0.85)	0.94 (0.89–0.98)	0.94 (0.90–0.99)
≤1h/day	74,961	1217	133.9	0.66 (0.62–0.70)	0.83 (0.78–0.89)	0.84 (0.79–0.90)
**Categories of computer use**						
≥4h/day (Reference)	19,253	515	213.6	1.00 (Reference)	1.00 (Reference)	1.00 (Reference)
2–3h/day	60,596	1719	226.9	0.92 (0.83–1.02)	1.01 (0.91–1.11)	1.00 (0.91–1.10)
≤1h/day	293,177	6951	187.6	0.78 (0.71–0.85)	1.00 (0.92–1.10)	0.99 (0.91–1.09)

^a^Model 1: Adjusted for no confounders

^b^Model 2: Adjusted for all confounders in Model 1 plus sex, body mass index (weight in kilograms/height in meters squared), smoking status (never, previous, current), employment (unemployed, employed), Townsend Deprivation Index (a numerical deprivation score generated based on employment, car ownership, home ownership and household overcrowding according to postcode of participants’ home address), alcohol consumption (never, previous, currently <3 times/week, currently ≥3 times/week), salt-adding behaviour (never/rarely, sometimes, usually, always), oily fish consumption (never, <once/week, once/week, >once/week), coffee intake (cups/day), fruit and vegetable intake (a composite score generated based on intake of fresh/dried fruit and intake of raw/cooked vegetable ranging from 0 to 4), processed/red meat intake (days/week), hypertension medication use, cholesterol-lowering medication use, glucose-lowering medication use, sleep (≤5, 6, 7, 8 and ≥9h/day) and moderate-to-vigorous physical activity (minutes/day)

^c^Model 3: Adjusted for all confounders in Model 2 plus the polygenic risk score, genotype array type and first ten principal components of genetic ancestry

**Fig. 1:**
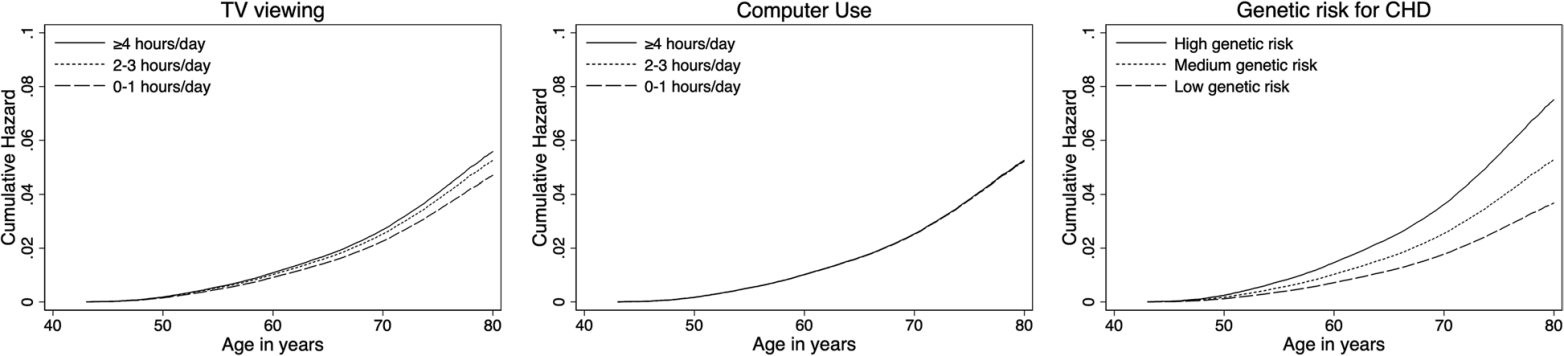
Cumulative hazard of incident coronary heart disease (CHD) for each category of TV viewing, computer use and genetic risk across age ranges. Cox regression models using age as the underlying timescale were adjusted for sex, body mass index (weight in kilograms/height in meters squared), smoking status (never, previous, current), employment (unemployed, employed), Townsend Deprivation Index (a numerical deprivation score generated based on employment, car ownership, home ownership and household overcrowding according to postcode of participants’ home address), alcohol consumption (never, previous, currently <3 times/week, currently ≥3 times/week), salt-adding behaviour (never/rarely, sometimes, usually, always), oily fish consumption (never, <once/week, once/week, >once/week), coffee intake (cups/day), fruit and vegetable intake (a composite score generated based on intake of fresh/dried fruit and intake of raw/cooked vegetable ranging from 0 to 4), processed/red meat intake (days/week), hypertension medication use, cholesterol-lowering medication use, glucose-lowering medication use, sleep (≤5, 6, 7, 8 and ≥9h/day), moderate-to-vigorous physical activity (minutes/day), polygenic risk scores, genotype array type and first ten principal components of genetic ancestry, with mutual adjustment of TV viewing and computer use in models using either TV viewing or computer use as the main exposure; and adjusted for sex, the genotype array type and first ten principal components of genetic ancestry in models using polygenic risk scores as the main exposure

Figure 1 shows the cumulative hazard of CHD for each category of TV viewing, computer use and genetic risk across age ranges. The cumulative hazard was consistently lower for ≤1h/day of TV viewing compared with ≥4h/day of TV viewing across all ages. The CHD hazards were highly similar between the three categories of computer use at all ages. Individuals at medium or high genetic risk for CHD had higher cumulative hazards of CHD than those at low genetic risk for CHD across all age ranges. The hazard ratios of CHD for medium and high genetic predispositions were 1.43 (95%CI 1.36–1.52) and 2.04 (95%CI 1.94–2.15), respectively, compared with those with low genetic predispositions.

**Fig. 2:**
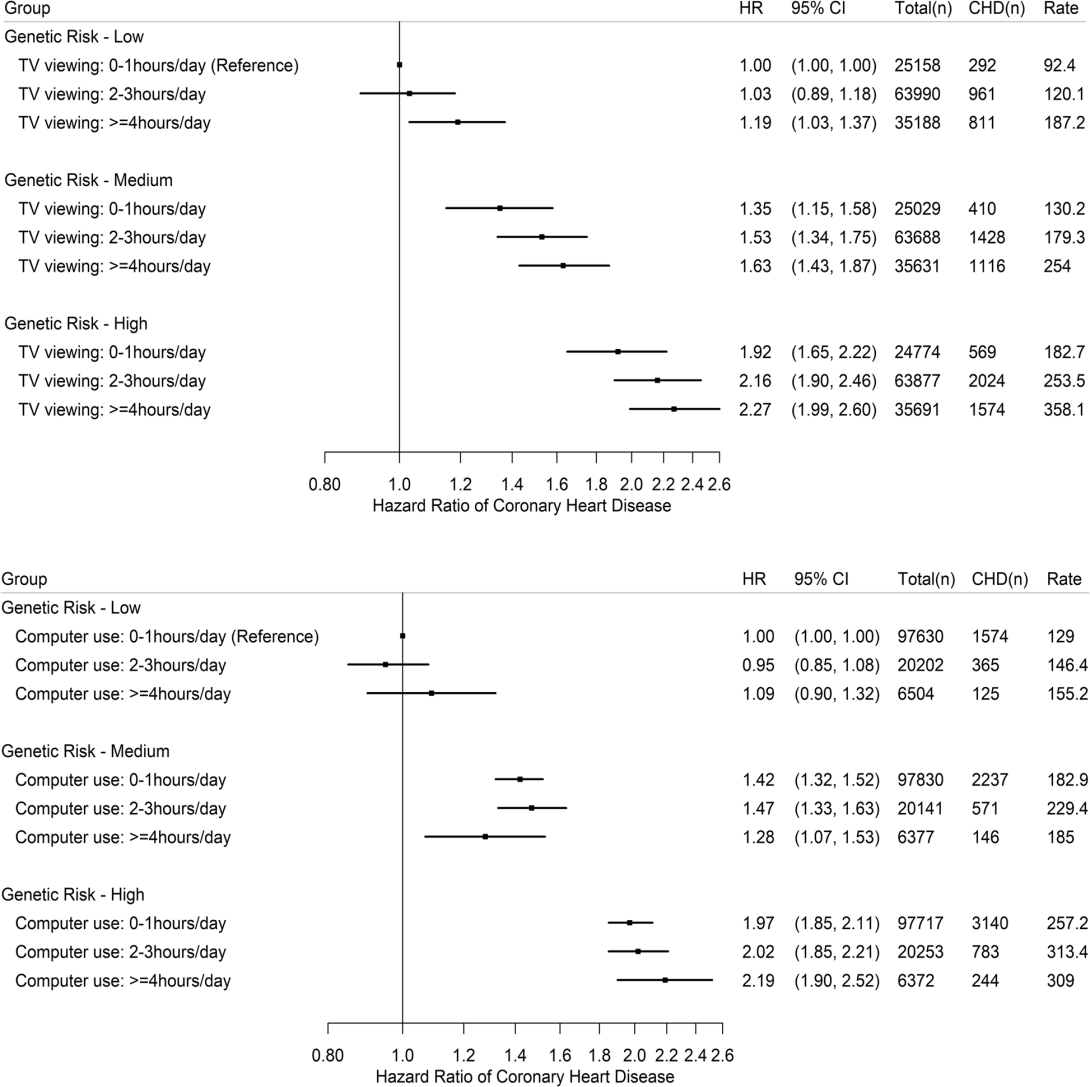
Joint associations of TV viewing (top panel) or computer use (bottom panel) and genetic risk with incident coronary heart disease (CHD). Cox regression models using age as the underlying timescale were adjusted for sex, body mass index (weight in kilograms/height in meters squared), smoking status (never, previous, current), employment (unemployed, employed), Townsend Deprivation Index (a numerical deprivation score generated based on employment, car ownership, home ownership and household overcrowding according to postcode of participants’ home address), alcohol consumption (never, previous, currently <3 times/week, currently ≥3 times/week), salt-adding behaviour (never/rarely, sometimes, usually, always), oily fish consumption (never, <once/week, once/week, >once/week), coffee intake (cups/day), fruit and vegetable intake (a composite score generated based on intake of fresh/dried fruit and intake of raw/cooked vegetable ranging from 0 to 4), processed/red meat intake (days/week), hypertension medication use, cholesterol-lowering medication use, glucose-lowering medication use, sleep (≤5, 6, 7, 8 and ≥9h/day), moderate-to-vigorous physical activity (minutes/day), genotype array type and first ten principal components of genetic ancestry, with mutual adjustment of the two exposure variables (TV viewing and computer use); no adjustment for the polygenic risk score. *P* values for multiplicative interactions between genetic risk and TV viewing and between genetic risk and computer use were 0.593 and 0.437, respectively. Rates are per 100,000 person-years. Abbreviations: HR hazard ratio, CHD coronary heart disease, CI confidence intervals

Figure 2 shows the joint associations of TV viewing, computer use and genetic risk with incident CHD. Relative to low genetic risk combined with ≤1h/day of TV viewing, low genetic risk combined with ≥4h/day of TV viewing was associated with a 19% (95%CI 3–37%) higher hazard of CHD. There was an increasing trend in hazard ratio values with higher genetic risk combined with the TV viewing categories, but watching TV for ≥4h/day was associated with higher CHD hazards compared with watching TV for ≤1h/day at medium genetic risk (hazard ratio: 1.21; 95%CI 1.07–1.37) as well as high genetic risk (hazard ratio: 1.19; 95%CI 1.07–1.32). No evidence of associations was found for any computer use category combined with any genetic risk stratum. There was no evidence of multiplicative interactions between genetic risk and TV viewing (p value:0.362) or computer use (p value:0.418) for incident CHD.

**Fig. 3:**
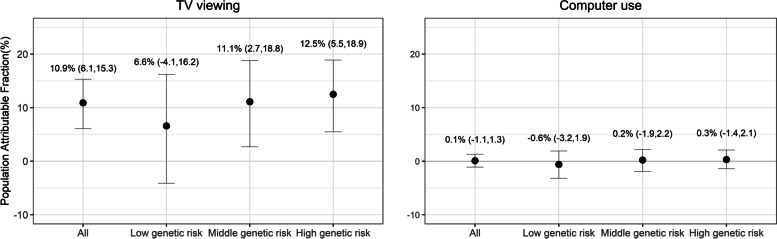
Population attributable fractions (PAF, %) indicating proportional risk reductions in coronary heart disease (CHD) that would be observed if ≥2h/day of TV viewing or computer use were reduced to ≤1h/day of TV viewing or computer use. Cox regression models using age as the underlying timescale were adjusted for sex, body mass index (weight in kilograms/height in meters squared), smoking status (never, previous, current), employment (unemployed, employed), Townsend Deprivation Index (a numerical deprivation score generated based on employment, car ownership, home ownership and household overcrowding according to postcode of participants’ home address), alcohol consumption (never, previous, currently <3 times/week, currently ≥3 times/week), salt-adding behaviour (never/rarely, sometimes, usually, always), oily fish consumption (never, <once/week, once/week, >once/week), coffee intake (cups/day), fruit and vegetable intake (a composite score generated based on intake of fresh/dried fruit and intake of raw/cooked vegetable ranging from 0 to 4), processed/red meat intake (days/week), hypertension medication use, cholesterol-lowering medication use, glucose-lowering medication use, sleep (≤5, 6, 7, 8 and ≥9h/day), moderate-to-vigorous physical activity (minutes/day), polygenic risk scores, genotype array type and first ten principal components of genetic ancestry, with mutual adjustment of the two exposure variables (TV viewing and computer use). Error bars indicate 95% confidence intervals

Figure 3 indicates PAF estimates for TV viewing and computer use overall and across genetic risk categories. Overall, 10.9% (95%CI 6.1–15.3%) of CHD could be averted if TV viewing were reduced from ≥2h/day to ≤1h/day, assuming causality. The PAF values were relatively larger for medium and high genetic risk than for low genetic risk, although the confidence intervals were wide and overlapping. The PAF values were much lower for computer use (Fig. 3).

## Discussion

This study using data from a large-scale cohort from the UK is the first to examine the interplay of specific types of screen-based sedentary activities and genetic susceptibility to CHD for incident CHD. Our study findings provide three major implications. First, TV viewing was positively associated with CHD risk, with a substantially lower risk observed for those watching TV for ≤1h/day. Importantly, this association was independent of individuals’ unique genetic susceptibility to CHD. This finding builds upon previous research that reported on strong positive associations of TV viewing time with the risk of cardiovascular events including CHD [11, 12]. Other research has also identified TV viewing as a predictor of cardiovascular disease [37] as well as intermediate cardiovascular risk markers [38]. However, our study is the first that recognises TV viewing as a strong independent risk marker of CHD when adjusting for genetic susceptibility to CHD as well as traditional risk markers.

Second, watching less TV was independently associated with lower CHD risk across strata of genetic risk including high genetic risk. This finding suggests that individuals whose genetic predisposition to CHD is high may have a lower risk of CHD merely when spending less time watching TV; TV viewing may be replaced by light-intensity physical activity, and reducing time spent in this behaviour may be relatively readily achieved than increasing the same amount of time spent in more structured, higher-intensity physical activity [39]. A few previous investigations found similar results, whereby the risk of cardiovascular events was lower in individuals (including those with high genetic risk) who adhered to a healthy lifestyle (typically defined as a combination of no smoking, no obesity, high physical activity and a healthy diet) [15–17] had higher levels of physical fitness and activity [18]. Similar to the present study, no evidence was identified for the interaction between genetic risk and adherence to a healthy lifestyle [15–17] or physical activity [18] (albeit evidence of interaction for physical fitness) [18] in previous research. Additional research is warranted to further explore the extent to which individuals with high genetic susceptibility would benefit from adopting different types of favourable lifestyle behaviours in comparison with those with low genetic susceptibility. Nonetheless, our study, in conjunction with previous research [15–18], reinforces the implementation of clinical trials aiming to prevent cardiovascular events through less time spent on TV viewing targeted at genetically susceptible individuals in the era of precision medicine [40].

Third, approximately 11% of CHD could be averted if TV viewing time were reduced from ≥2h/day to ≤1h/day, even after accounting for genetic risk, assuming causality [10]. The reduction in CHD may be relatively larger in a population with higher genetic susceptibility, despite the wide and overlapping confidence intervals. Nonetheless, these results are consistent with findings of previous research [15, 16] which also indicated larger absolute CHD-risk reductions for a given decrease in risk behaviour in more genetically susceptible individuals. Additional gene-environment interaction research estimating absolute risk in individuals of different genetic make-ups is warranted to further confirm this observation. However, future clinical trials comparing the effects of reducing TV viewing time between individuals at high versus low genetic risk will provide insights into the utilization of genotype information lifestyle modification from a CHD prevention perspective [41].

In contrast to the findings for TV viewing, we found no evidence of associations for computer use. This observation corroborates existing evidence that TV viewing, but not computer use, is positively associated with markers of cardiometabolic risk [42]. Potential explanations include higher reliability of recalling TV viewing (i.e., lower measurement error) compared with recalling computer use [43, 44] and slightly lower amounts of energy expended while watching TV than using a computer [45]. Another explanation is unhealthy snacking behaviours that may have occurred while watching TV in some of the participants, thereby leading to stronger associations for TV viewing [46], but our models for TV viewing and computer use were adjusted for multiple diet-behavior variables. Furthermore, TV viewing tends to occur in a more prolonged, uninterrupted manner, particularly in the evening time after dinner [47], leading to elevated levels of postprandial glucose and lipid (i.e. intermediate metabolic risk markers but not acting as confounders) [48, 49].

### Limitations

This study has several limitations. First, due to the observational nature of this study, conclusions about causality cannot be drawn. As such, the PAF analyses were grounded upon an assumption that the associations of TV viewing and computer use with CHD risk are causal [10], which has yet to be fully determined in the current literature. Moreover, there may be a measurement error due to recall bias in assessing TV viewing and computer use through questionnaires. Larger measurement errors in TV viewing and computer use may have led to attenuated associations with CHD [50]. There is also potential for residual confounding due to unmeasured confounders (e.g. unhealthy eating behaviours while watching TV) or measurement error in the self-reported confounders (e.g. smoking, employment, socioeconomic status, alcohol consumption, food intake variables, medication use, sleep and moderate-to-vigorous physical activity). Furthermore, we included only two prevalent forms of screen-based sedentary activities, due to the limited information on other types of sedentary behaviour available in the UK Biobank database. Hence, our findings may not be applicable to overall sedentary/sitting time as well as other types of sedentary behaviour [10]. Given that this study is of European descendants, findings may not be generalisable to individuals of other ethnicities. Moreover, UK Biobank participants tend to have more favourable health profiles than the general UK population [51], so generalizing our findings to average UK adults or individuals with sub-clinical symptoms should be made with caution. While we excluded the first 2 years and 4 years of follow-up in the main and sensitivity analyses, respectively, there may still be potential for reverse causality in the associations identified herein.

## Conclusions

TV viewing is a strong risk marker of CHD, independently of genetic susceptibility. Higher genetic risk for CHD is also associated with a higher risk of developing CHD. Lower TV viewing time is associated with lower CHD incidence across all strata of genetic risk, with somewhat stronger associations at the higher genetic susceptibility. Clinical trials customised to individuals whose genetic susceptibility is high should consider reducing TV viewing a behavioural target for the prevention of cardiovascular events.

## Supplementary Information

**Additional file 1: Figure S1.** A participant flow chart. **Figure S2.** Distribution of the calculated polygenic risk score (PRS) for coronary heart disease using 300 uncorrelated SNPs. **Figure S3.** Cubic spline models representing trends of associations between continuous variables of TV viewing (relative to 1 hour/day of TV viewing), computer use (relative to 1 hour/day of computer use) and polygenic risk score (relative to a polygenic risk score of 18) and incident coronary heart disease (CHD). **Figure S4.** Distribution of the calculated polygenic risk score (PRS) for coronary heart disease using 46 SNPs were genome-wide significant at a p-value of 5×10^-8^ and in low linkage disequilibrium defined according to r^2^<0.001. **Table S1.** A list of 300 Single-Nucleotide Polymorphisms (SNPs) known to be associated with coronary heart disease risk. **Table S2.** Associations of TV viewing and computer use with incident coronary heart disease (CHD) after excluding an additional two years of follow-up. **Table S3.** Associations of TV viewing and computer use with incident coronary heart disease (CHD) after excluding individuals with poor self-reported health status (i.e. based on the 4-level self-reported health ratings; poor [excluded], fair, good, excellent). **Table S4.** Associations of TV viewing and computer use with incident coronary heart disease (CHD) after excluding individuals with 2nd-degree genetic relatedness. **Table S5.** Associations of genetic risk for coronary heart disease and TV viewing and computer use with incident coronary heart disease (CHD) using a weighted polygenic risk score calculated based only on 46 lead SNPs (from 46 loci) which were genome-wide significant at a p-value of 5×10^-8^ and in low linkage disequilibrium defined according to r^2^<0.001. **Table S6.** Associations of TV viewing and computer use with incident coronary heart disease (CHD) using values imputed for the covariates missing, assuming data missing at random. **Table S7.** Associations of TV viewing and computer use with incident coronary heart disease (CHD) including prevalence of type 2 diabetes and renal dysfunction as potential confounders. **Table S8.** Associations of TV viewing and computer use with incident coronary heart disease (CHD) excluding body mass index as a potential confounder. **Table S9.** Associations of TV viewing and computer use with incident coronary heart disease (CHD) using CHD follow-up censored on January 1st, 2020 to account for potential CHD cases not captured due to participants’ fear of visiting clinics during COVID-19. **Table S10.** Associations of TV viewing and computer use with incident coronary heart disease (CHD) with adjustment for education, household income and occupation as individual-level indicators of socio-economic status as opposed to area-level socio-economic status (Townsend Deprivation Index).

## Data Availability

All data from the UK Biobank project are publicly available upon data access application by researchers through the UK Biobank website (https://www.ukbiobank.ac.uk/).

## Abbreviations

BMI: Body mass index
CHD: Coronary heart disease
GWAS: Genome-Wide Association Study
ICD: International Classification of Diseases
PAFs: Population attributable fractions
SNPs: Single-nucleotide polymorphisms
TV: Television
