# Supplementary material for: Genetic susceptibility, screen-based sedentary activities and incidence of coronary heart disease
Source: BMC Med. 2022 May 24;20:188. doi: 10.1186/s12916-022-02380-7 (PMC9126635; doi:10.1186/s12916-022-02380-7)
Supplement: Supplementary file 1 — Additional file 1: Figure S1. A participant flow chart. Figure S2. Distribution of the calculated polygenic risk score (PRS) for coronary heart disease using 300 uncorrelated SNPs. Figure S3. Cubic spline models representing trends of associations between continuous variables of TV viewing (relative to 1 hour/day of TV viewing), computer use (relative to 1 hour/day of computer use) and polygenic risk score (relative to a polygenic risk score of 18) and incident coronary heart disease (CHD). Figure S4. Distribution of the calculated polygenic risk score (PRS) for coronary heart disease using 46 SNPs were genome-wide significant at a p-value of 5×10^-8^ and in low linkage disequilibrium defined according to r2<0.001. Table S1. A list of 300 Single-Nucleotide Polymorphisms (SNPs) known to be associated with coronary heart disease risk. Table S2. Associations of TV viewing and computer use with incident coronary heart disease (CHD) after excluding an additional two years of follow-up. Table S3. Associations of TV viewing and computer use with incident coronary heart disease (CHD) after excluding individuals with poor self-reported health status (i.e. based on the 4-level self-reported health ratings; poor [excluded], fair, good, excellent). Table S4. Associations of TV viewing and computer use with incident coronary heart disease (CHD) after excluding individuals with 2nd-degree genetic relatedness. Table S5. Associations of genetic risk for coronary heart disease and TV viewing and computer use with incident coronary heart disease (CHD) using a weighted polygenic risk score calculated based only on 46 lead SNPs (from 46 loci) which were genome-wide significant at a p-value of 5×10^-8^ and in low linkage disequilibrium defined according to r2<0.001. Table S6. Associations of TV viewing and computer use with incident coronary heart disease (CHD) using values imputed for the covariates missing, assuming data missing at random. Table S7. Associations of TV viewing and computer [file 12916_2022_2380_MOESM1_ESM.docx]

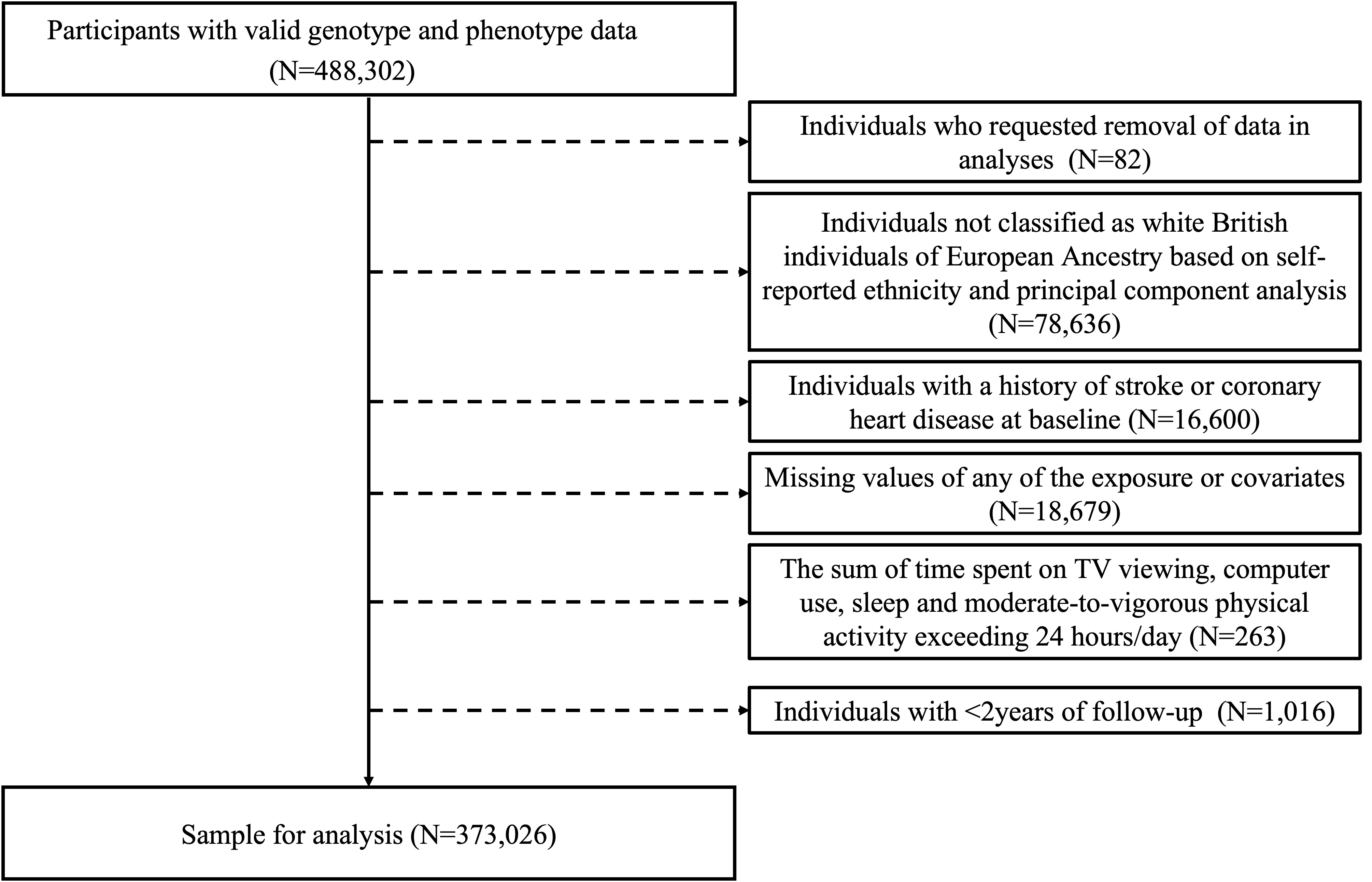


Supplemental Figure 1. A participant flow chart.


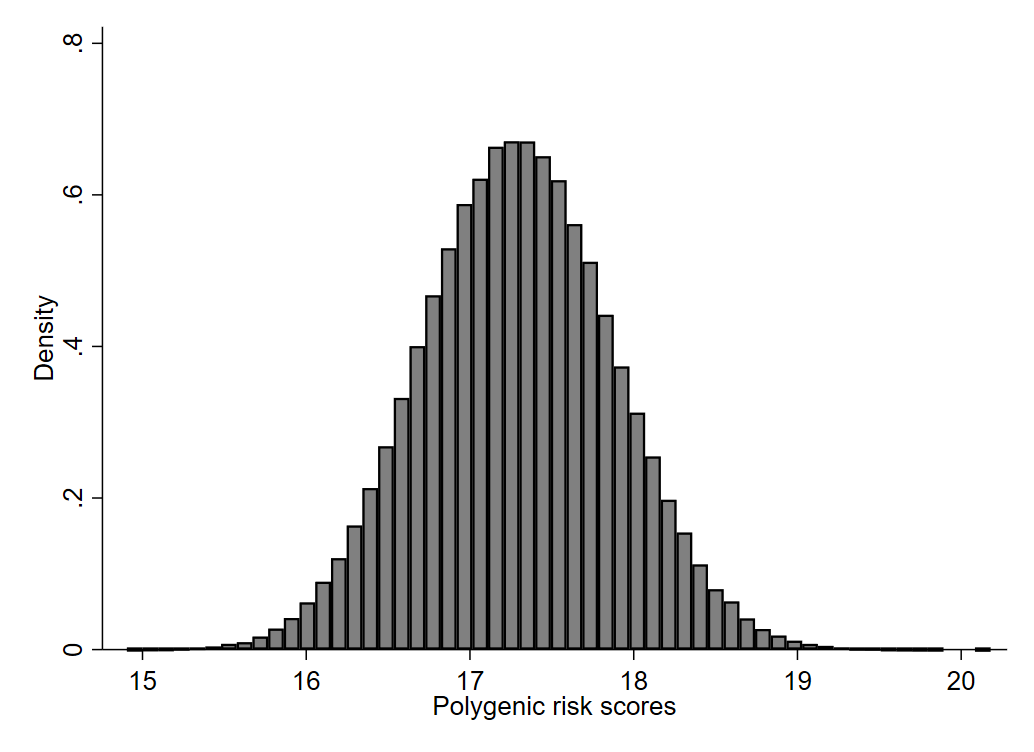


Supplemental Figure 2. Distribution of the calculated polygenic risk score (PRS) for coronary heart disease using 300 uncorrelated SNPs.


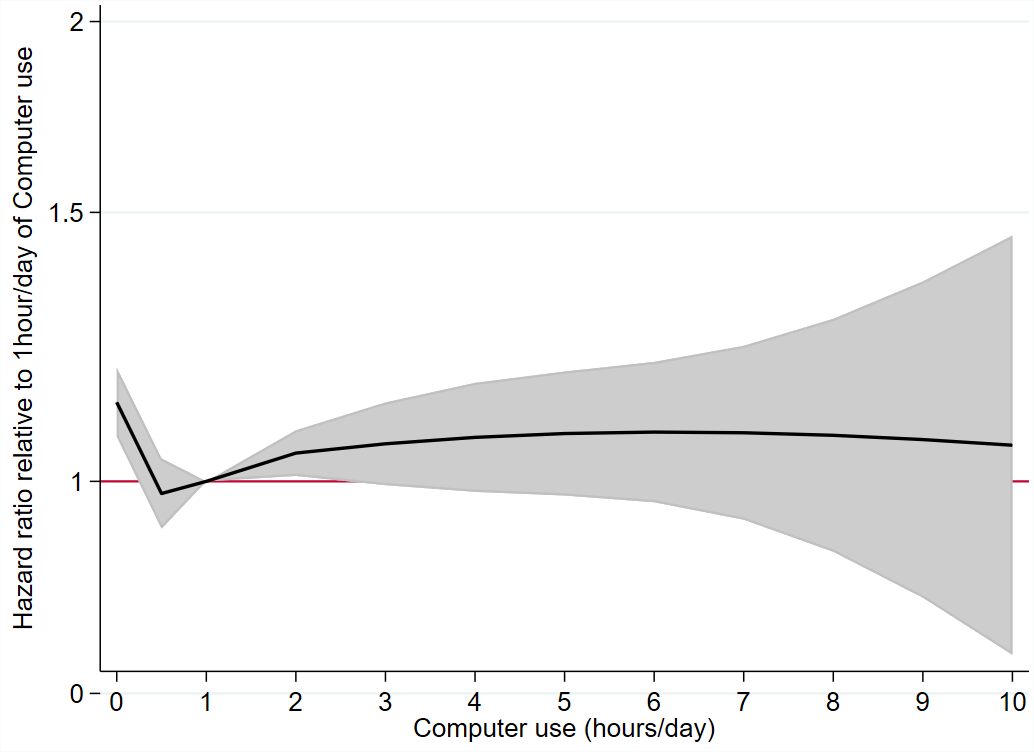

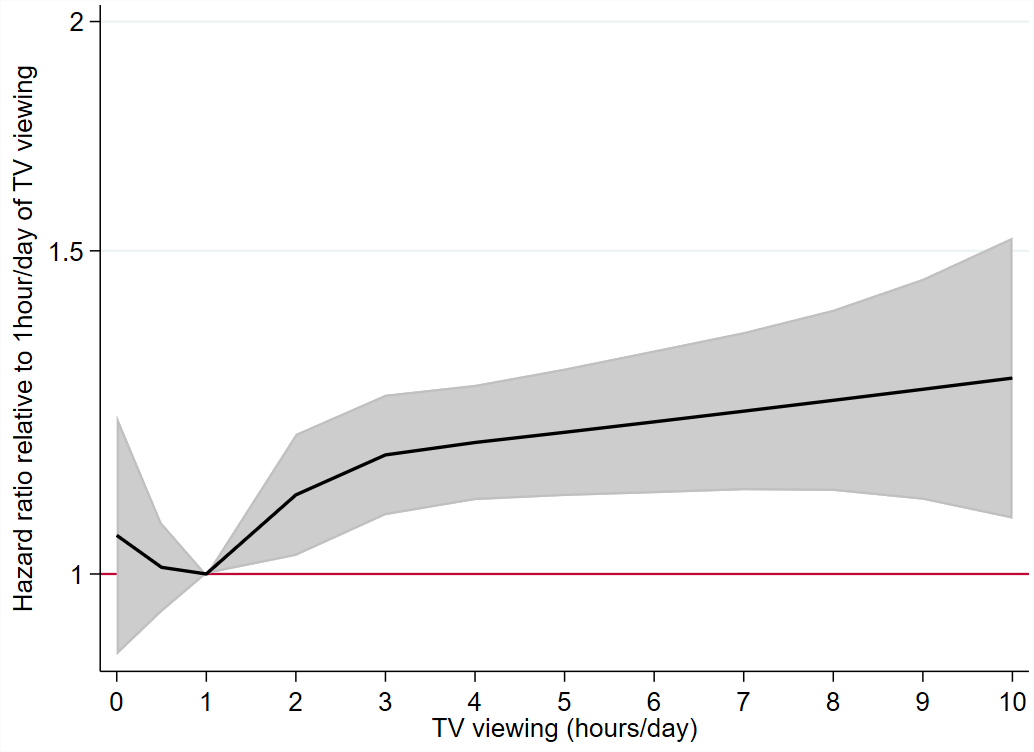

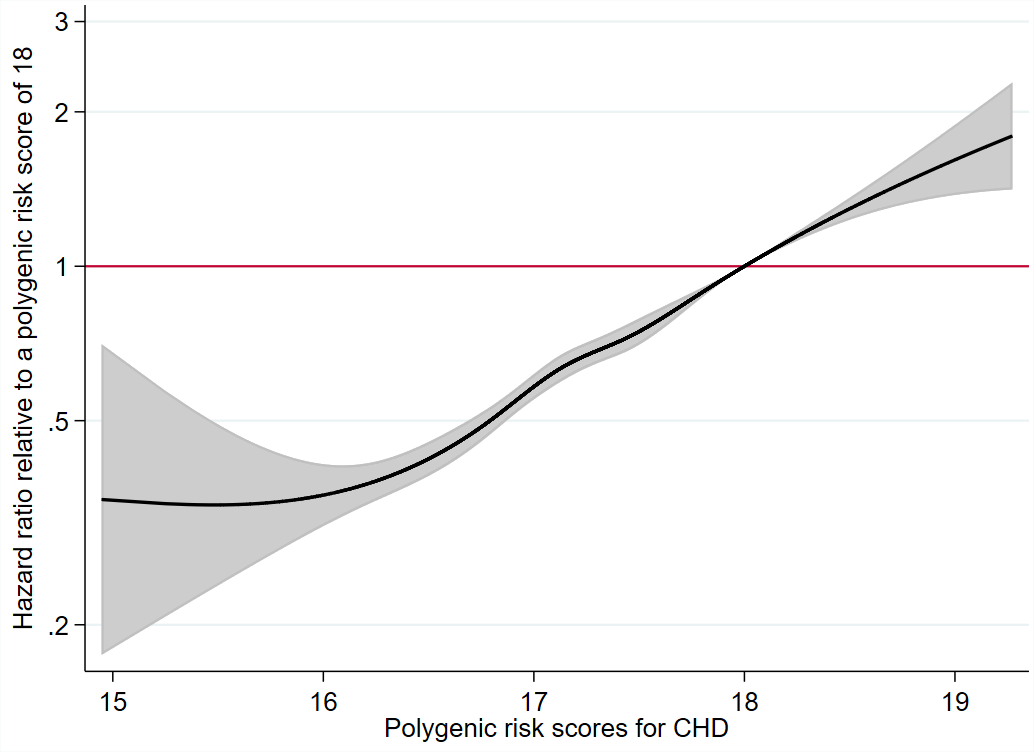


Supplemental Figure 3. Cubic spline models representing trends of associations between continuous variables of TV viewing (relative to 1 hour/day of TV viewing), computer use (relative to 1 hour/day of computer use) and polygenic risk score (relative to a polygenic risk score of 18) and incident coronary heart disease (CHD). Cox regression models using age as the underlying timescale and with five equally spaced knots were adjusted for sex, body mass index (weight in kilograms/height in meters squared), smoking status (never, previous, current), employment (unemployed, employed), Townsend Deprivation Index (a numerical deprivation score generated based on employment, car ownership, home ownership and household overcrowding according to postcode of participants’ home address), alcohol consumption (never, previous, currently <3 times/week, currently ≥3 times/week), salt-adding behaviour (never/rarely, sometimes, usually, always), oily fish consumption (never, <once/week, once/week, >once/week), coffee intake (cups/day), fruit and vegetable intake (a composite score generated based on intake of fresh/dried fruit and intake of raw/cooked vegetable ranging from 0 to 4), processed/red meat intake (days/week), hypertension medication use, cholesterol-lowering medication use, glucose-lowering medication use, sleep (≤5, 6, 7, 8 and ≥9hours/day), moderate-to-vigorous physical activity (minutes/day), genotype array type and first ten principal components of genetic ancestry, with mutual adjustment of TV viewing and computer use in models using either TV viewing or computer use as the main exposure; and adjusted for sex, the genotype array type and first ten principal components of genetic ancestry in models using polygenic risk scores as the main exposure.


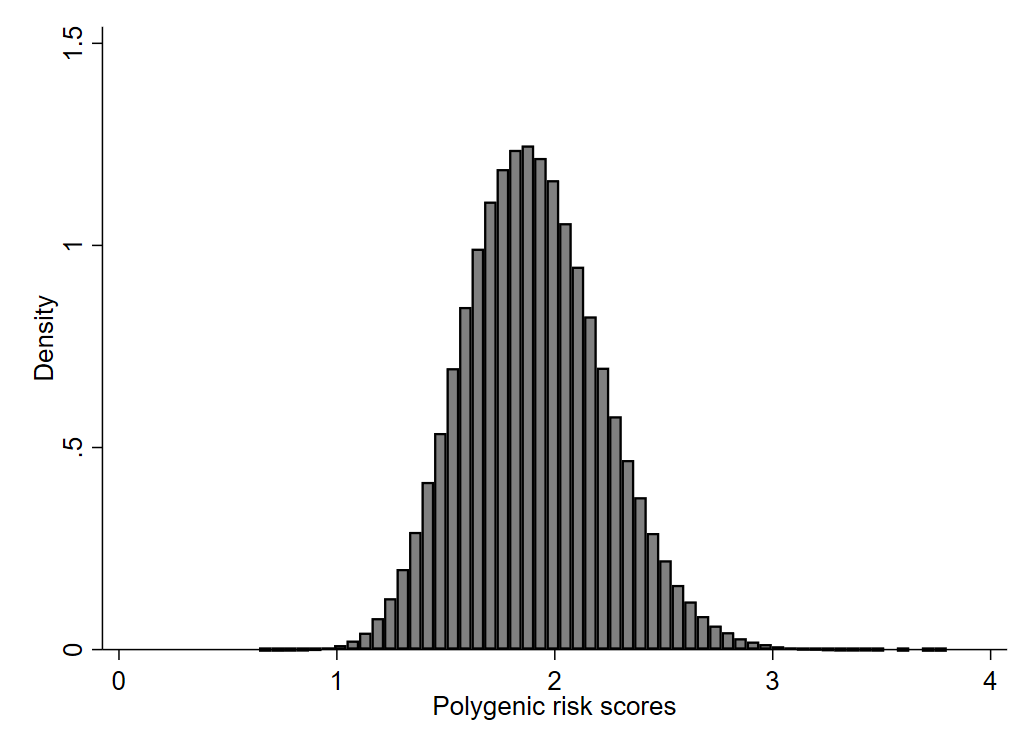


Supplemental Figure 4. Distribution of the calculated polygenic risk score (PRS) for coronary heart disease using 46 SNPs were genome-wide significant at a p-value of 5×10^-8^ and in low linkage disequilibrium defined according to r^2^<0.001.

Supplemental Table 1. A list of 300 Single-Nucleotide Polymorphisms (SNPs) known to be associated with coronary heart disease risk.

| **SNPs** | **Effect allele** | **Other allele** | **Beta** | **P value** |
| --- | --- | --- | --- | --- |
| rs2843152 | C | G | 0.042 | 1.34E-05 |
| rs35465346 | G | A | 0.055 | 5.91E-06 |
| rs7538207 | C | T | 0.098 | 5.73E-06 |
| rs11811081 | C | A | 0.077 | 5.65E-05 |
| rs12733730 | A | G | 0.046 | 1.79E-05 |
| rs11485595 | T | C | 0.04 | 4.00E-06 |
| rs34232196 | C | T | 0.055 | 2.87E-08 |
| rs11591147^#^ | G | T | 0.221 | 2.84E-10 |
| rs17111652 | T | C | 0.082 | 1.34E-05 |
| rs6665249 | A | G | 0.042 | 1.54E-05 |
| rs56170783^#^ | A | C | 0.104 | 2.14E-12 |
| rs2149821 | A | T | 0.039 | 3.17E-05 |
| rs10890013 | T | C | 0.035 | 2.26E-05 |
| rs113832197 | T | C | 0.081 | 3.40E-05 |
| rs7528419^#^ | A | G | 0.109 | 3.77E-27 |
| rs11552449 | T | C | 0.045 | 3.90E-05 |
| rs10305649 | A | C | 0.114 | 2.87E-06 |
| rs11810571 | G | C | 0.058 | 2.21E-08 |
| rs6689306^#^ | A | G | 0.05 | 1.46E-09 |
| rs2789422 | G | A | 0.035 | 4.63E-05 |
| rs6413828 | A | T | 0.038 | 1.77E-05 |
| rs183692864 | G | A | 0.244 | 5.49E-05 |
| rs1892094 | C | T | 0.02 | 1.55E-02 |
| rs6700559 | C | T | 0.022 | 6.96E-03 |
| rs2820315 | T | C | 0.043 | 2.09E-06 |
| rs67180937^#^ | G | T | 0.071 | 8.45E-14 |
| rs17464857 | T | G | 0.06 | 6.90E-07 |
| rs3755549 | C | T | 0.034 | 2.64E-05 |
| rs2709437 | T | C | 0.034 | 4.38E-05 |
| rs16986953^#^ | A | G | 0.105 | 4.77E-10 |
| rs585967^#^ | C | A | 0.066 | 2.76E-08 |
| rs58560619 | C | T | 0.036 | 1.65E-05 |
| rs4299376^#^ | G | T | 0.055 | 5.65E-10 |
| rs4076834 | T | G | 0.1 | 1.23E-08 |
| rs139591697 | T | C | 0.102 | 5.15E-05 |
| rs72375964 | A | G | 0.035 | 5.01E-05 |
| rs7568458^#^ | A | T | 0.061 | 2.39E-13 |
| rs149366039 | T | C | 0.731 | 4.03E-05 |
| rs7578433 | T | C | 0.07 | 3.87E-05 |
| rs79716828 | C | A | 0.111 | 8.63E-06 |
| rs6761276 | T | C | 0.036 | 2.66E-05 |
| rs7570006 | C | T | 0.048 | 3.78E-05 |
| rs17678683 | G | T | 0.077 | 1.15E-07 |
| rs35500812 | A | C | 0.042 | 3.20E-06 |
| rs12619842 | G | C | 0.048 | 8.57E-06 |
| rs62172372 | A | G | 0.046 | 3.61E-05 |
| rs114123510^#^ | A | T | 0.118 | 2.88E-19 |
| rs2011559 | G | A | 0.054 | 2.44E-05 |
| rs1250229^#^ | T | C | 0.069 | 1.85E-13 |
| rs2161967 | T | G | 0.039 | 6.21E-06 |
| rs2972146 | T | G | 0.047 | 6.50E-08 |
| rs10168194 | C | G | 0.039 | 6.78E-06 |
| rs13003675 | T | C | 0.042 | 1.72E-06 |
| rs10929113 | C | T | 0.042 | 5.24E-05 |
| rs143803699 | G | C | 0.12 | 2.95E-05 |
| rs748431 | G | T | 0.041 | 9.04E-07 |
| rs3821396 | G | A | 0.06 | 6.57E-06 |
| rs7623687^#^ | A | C | 0.072 | 3.72E-09 |
| rs77622129 | A | G | 0.094 | 2.00E-05 |
| rs62253653 | A | G | 0.038 | 4.35E-05 |
| rs71331765 | G | C | 0.051 | 4.47E-05 |
| rs6787409 | C | T | 0.039 | 1.63E-05 |
| rs9818870^#^ | T | C | 0.068 | 7.82E-09 |
| rs4632520 | C | T | 0.038 | 2.90E-05 |
| rs12493885^#^ | C | G | 0.071 | 3.29E-08 |
| rs10513507 | C | T | 0.036 | 2.20E-05 |
| rs34229028 | G | A | 0.037 | 1.25E-05 |
| rs9869263 | G | A | 0.048 | 3.74E-05 |
| rs113148244 | G | T | 0.144 | 2.20E-05 |
| rs16994919 | A | G | 0.06 | 9.55E-06 |
| rs2616407 | C | T | 0.049 | 5.60E-06 |
| rs13134452 | C | T | 0.037 | 3.16E-05 |
| rs72627509 | G | C | 0.054 | 8.10E-08 |
| rs10857147^#^ | T | A | 0.054 | 8.96E-09 |
| rs138495951 | G | A | 0.16 | 3.84E-05 |
| rs7678555 | C | A | 0.048 | 1.43E-07 |
| rs144059514 | G | A | 0.085 | 4.50E-03 |
| rs13109172 | C | T | 0.038 | 5.73E-06 |
| rs4593108 | C | G | 0.058 | 1.95E-08 |
| rs6841581^#^ | A | G | 0.068 | 4.57E-10 |
| rs7435973 | G | A | 0.059 | 4.51E-07 |
| rs3796587^#^ | C | G | 0.063 | 1.24E-09 |
| rs869396 | C | A | 0.039 | 1.85E-06 |
| rs11728590 | G | T | 0.037 | 1.97E-05 |
| rs71600236 | C | G | 0.039 | 6.54E-06 |
| rs112941079 | A | G | 0.059 | 3.86E-06 |
| rs5868014 | G | A | 0.052 | 1.04E-06 |
| rs111777100 | A | G | 0.093 | 5.35E-05 |
| rs288187 | C | T | 0.046 | 2.63E-05 |
| rs1800449 | T | C | 0.056 | 4.06E-07 |
| rs1500187 | G | A | 0.037 | 9.73E-06 |
| rs6883598 | C | A | 0.039 | 2.32E-05 |
| rs273909 | G | A | 0.053 | 9.94E-04 |
| rs251023 | G | A | 0.038 | 8.34E-06 |
| rs11955380 | C | A | 0.06 | 1.28E-05 |
| rs3776307 | G | A | 0.038 | 9.40E-06 |
| rs6860540 | G | A | 0.035 | 5.49E-05 |
| rs9501744 | C | T | 0.064 | 1.08E-06 |
| rs421329 | C | T | 0.049 | 1.55E-06 |
| rs742115 | C | T | 0.036 | 2.86E-05 |
| rs6458138 | G | A | 0.065 | 6.13E-05 |
| rs9349379^#^ | G | A | 0.105 | 9.95E-36 |
| rs13200993^#^ | T | C | 0.05 | 5.60E-09 |
| rs3130683 | T | C | 0.077 | 2.77E-08 |
| rs9268402 | A | G | 0.014 | 1.28E-01 |
| rs4472337 | T | C | 0.055 | 2.42E-06 |
| rs17609940 | G | C | 0.029 | 7.11E-03 |
| rs56015508 | C | A | 0.054 | 1.08E-07 |
| rs1214752 | C | T | 0.039 | 3.26E-06 |
| rs6905288 | A | G | 0.039 | 3.26E-06 |
| rs1330633 | G | A | 0.067 | 5.89E-05 |
| rs194937 | A | G | 0.048 | 1.26E-05 |
| rs11153071 | G | A | 0.05 | 3.00E-06 |
| rs9398803 | A | G | 0.034 | 6.23E-05 |
| rs12202017^#^ | A | G | 0.066 | 6.02E-14 |
| rs9493752 | A | G | 0.154 | 7.05E-06 |
| rs2492304 | A | T | 0.033 | 5.59E-05 |
| rs2153219 | A | G | 0.051 | 2.59E-06 |
| rs55730499^#^ | T | C | 0.268 | 5.64E-49 |
| rs186696265 | T | C | 0.466 | 8.97E-36 |
| rs4252198 | G | C | 0.152 | 1.66E-05 |
| rs79018195 | C | T | 0.304 | 1.26E-06 |
| rs41269133 | T | C | 0.079 | 4.78E-08 |
| rs9364552 | C | G | 0.035 | 1.50E-05 |
| rs6956990 | C | T | 0.115 | 9.11E-06 |
| rs11509880 | A | G | 0.036 | 2.11E-05 |
| rs2107595^#^ | A | G | 0.074 | 3.41E-13 |
| rs55889159 | A | C | 0.035 | 5.11E-05 |
| rs78850423 | A | G | 0.143 | 9.49E-06 |
| rs2971672 | C | A | 0.036 | 1.90E-05 |
| rs1088868 | G | A | 0.04 | 3.84E-05 |
| rs35146811 | C | A | 0.042 | 5.36E-06 |
| rs112370447 | T | C | 0.045 | 9.62E-07 |
| rs10953541 | C | T | 0.027 | 5.77E-03 |
| rs2024233 | G | A | 0.037 | 2.85E-05 |
| rs11556924^#^ | C | T | 0.067 | 6.26E-13 |
| rs2286198 | G | A | 0.051 | 3.42E-07 |
| rs3918226^#^ | T | C | 0.125 | 1.58E-12 |
| rs2083636 | T | G | 0.051 | 6.44E-08 |
| rs28597716 | A | G | 0.051 | 6.85E-06 |
| rs16885577 | G | A | 0.049 | 2.56E-05 |
| rs10109493 | A | G | 0.061 | 4.93E-05 |
| rs72658939 | G | C | 0.046 | 2.80E-05 |
| rs77211063 | T | C | 0.11 | 2.30E-05 |
| rs10955380 | C | A | 0.04 | 1.19E-05 |
| rs2954029^#^ | A | T | 0.06 | 5.24E-13 |
| rs117938894 | G | A | 0.161 | 3.73E-05 |
| rs75824083 | C | T | 0.907 | 5.09E-05 |
| rs58594043 | A | G | 0.051 | 3.14E-05 |
| rs34914400 | T | C | 0.813 | 1.60E-05 |
| rs2891168^#^ | G | A | 0.173 | 5.23E-104 |
| rs3217992 | T | C | 0.122 | 5.50E-49 |
| rs1333050 | T | C | 0.124 | 2.39E-41 |
| rs4149311 | T | C | 0.052 | 9.06E-06 |
| rs1967604 | A | G | 0.037 | 5.01E-05 |
| rs111245230 | C | T | 0.109 | 8.29E-07 |
| rs781622 | T | C | 0.036 | 2.13E-05 |
| rs77275410 | C | T | 0.061 | 2.55E-05 |
| rs10818583 | A | G | 0.043 | 4.85E-06 |
| rs507666^#^ | A | G | 0.074 | 1.34E-12 |
| rs11257613 | G | A | 0.035 | 1.82E-05 |
| rs7094201 | G | A | 0.062 | 5.77E-05 |
| rs1887318^#^ | T | C | 0.058 | 4.12E-12 |
| rs1870634^#^ | G | T | 0.062 | 5.51E-13 |
| rs1657345 | A | G | 0.081 | 4.58E-12 |
| rs17726488 | T | C | 0.103 | 1.01E-05 |
| rs4691 | T | C | 0.041 | 8.67E-06 |
| rs7098414 | A | C | 0.046 | 2.66E-06 |
| rs2246942^#^ | G | A | 0.076 | 3.51E-16 |
| rs59898454 | A | G | 0.147 | 2.09E-06 |
| rs11191416^#^ | T | G | 0.073 | 5.58E-09 |
| rs12252333 | G | A | 0.047 | 4.90E-06 |
| rs2257129 | C | T | 0.096 | 1.64E-06 |
| rs2281674 | C | G | 0.064 | 4.68E-05 |
| rs28596486 | C | T | 0.052 | 2.91E-06 |
| rs56210063 | C | G | 0.068 | 4.00E-05 |
| rs10840293^#^ | A | G | 0.049 | 6.88E-09 |
| rs11042937 | T | G | 0.011 | 2.02E-01 |
| rs3993105 | T | C | 0.047 | 1.06E-07 |
| rs11462682 | G | A | 0.044 | 8.35E-05 |
| rs146039567 | C | A | 0.149 | 2.20E-05 |
| rs2306029 | T | C | 0.039 | 1.57E-05 |
| rs2727020 | C | G | 0.042 | 4.70E-06 |
| rs12146487 | G | A | 0.048 | 2.28E-05 |
| rs12801636 | G | A | 0.043 | 7.75E-06 |
| rs571353 | C | T | 0.043 | 3.47E-06 |
| rs634552 | G | T | 0.05 | 4.71E-05 |
| rs3133293 | G | T | 0.041 | 3.34E-06 |
| rs17712139 | G | A | 0.041 | 3.62E-05 |
| rs2212437 | A | G | 0.04 | 7.63E-06 |
| rs2839812^#^ | T | A | 0.06 | 1.99E-11 |
| rs567040 | C | T | 0.037 | 6.21E-05 |
| rs964184 | G | C | 0.051 | 4.68E-06 |
| rs3782774 | G | A | 0.036 | 2.17E-05 |
| rs3861086 | C | T | 0.045 | 6.34E-07 |
| rs11170820 | G | C | 0.089 | 2.38E-07 |
| rs56245751 | T | C | 0.061 | 1.13E-05 |
| rs11172113 | C | T | 0.036 | 2.44E-05 |
| rs2229357 | G | A | 0.047 | 3.39E-06 |
| rs6538176 | T | C | 0.047 | 6.24E-06 |
| rs11115214 | C | T | 0.042 | 3.71E-05 |
| rs2681472^#^ | G | A | 0.066 | 7.63E-11 |
| rs10774625^#^ | A | G | 0.064 | 9.22E-14 |
| rs2244608^#^ | G | A | 0.051 | 2.32E-09 |
| rs11057401 | T | A | 0.044 | 1.32E-06 |
| rs11057830 | A | G | 0.069 | 4.24E-09 |
| rs1924981 | T | C | 0.046 | 1.86E-07 |
| rs9591012 | G | A | 0.038 | 1.87E-05 |
| rs73468973 | A | G | 0.043 | 5.68E-05 |
| rs75535189 | C | T | 0.262 | 1.01E-05 |
| rs9515203^#^ | T | C | 0.062 | 6.48E-10 |
| rs4773141 | G | C | 0.059 | 9.46E-10 |
| rs9588107 | A | G | 0.033 | 7.80E-05 |
| rs12867664 | A | G | 0.086 | 1.57E-05 |
| rs17102313 | T | C | 0.615 | 4.64E-05 |
| rs12891473 | C | T | 0.035 | 2.33E-05 |
| rs4506804 | T | G | 0.034 | 3.14E-05 |
| rs3832966 | C | T | 0.037 | 4.67E-06 |
| rs112635299 | G | T | 0.163 | 1.65E-05 |
| rs10139550^#^ | G | C | 0.051 | 1.84E-09 |
| rs113025579 | C | T | 0.125 | 4.94E-05 |
| rs147580454 | C | T | 0.033 | 9.72E-05 |
| rs6494488 | A | G | 0.034 | 1.80E-03 |
| rs72743461^#^ | C | A | 0.071 | 4.81E-12 |
| rs7164479^#^ | T | C | 0.072 | 6.38E-18 |
| rs2083460 | T | C | 0.072 | 1.41E-07 |
| rs2071382^#^ | T | C | 0.062 | 7.14E-13 |
| rs17581137 | A | C | 0.042 | 1.38E-05 |
| rs116082507 | T | C | 1.204 | 4.13E-06 |
| rs7185993 | T | C | 0.036 | 1.23E-05 |
| rs247616 | C | T | 0.044 | 1.01E-06 |
| rs35259348 | C | G | 0.051 | 1.15E-07 |
| rs1050362 | A | C | 0.029 | 4.84E-04 |
| rs9929108 | T | G | 0.047 | 2.32E-07 |
| rs3851738 | C | G | 0.041 | 6.67E-07 |
| rs7500448^#^ | A | G | 0.059 | 5.14E-09 |
| rs1968266 | T | C | 0.037 | 5.23E-05 |
| rs117592425 | A | C | 0.203 | 4.14E-06 |
| rs113348108 | G | A | 0.044 | 2.02E-07 |
| rs8068571 | T | C | 0.042 | 1.76E-05 |
| rs9897596 | T | C | 0.039 | 3.13E-06 |
| rs13723 | G | A | 0.035 | 2.39E-05 |
| rs148720362 | C | T | 0.035 | 5.06E-04 |
| rs1122326 | C | A | 0.05 | 2.76E-06 |
| rs8068844 | C | T | 0.043 | 3.93E-07 |
| rs17608766 | C | T | 0.044 | 6.07E-04 |
| rs46522 | T | C | 0.033 | 9.31E-05 |
| rs4643373 | T | C | 0.046 | 1.20E-06 |
| rs62076439 | T | G | 0.044 | 7.83E-07 |
| rs8068952^#^ | G | C | 0.07 | 1.41E-09 |
| rs7212798 | C | T | 0.063 | 4.37E-08 |
| rs6504218 | G | A | 0.041 | 9.41E-07 |
| rs11077501 | C | T | 0.037 | 2.19E-05 |
| rs75589791 | G | A | 0.065 | 4.58E-05 |
| rs35489971 | A | G | 0.054 | 1.06E-06 |
| rs11654510 | C | A | 0.058 | 9.46E-06 |
| rs7211674 | C | A | 0.034 | 6.14E-05 |
| rs9951447 | C | T | 0.038 | 4.37E-06 |
| rs178002 | G | A | 0.042 | 1.91E-06 |
| rs12922 | A | C | 0.05 | 2.92E-05 |
| rs833509 | C | T | 0.039 | 2.58E-05 |
| rs948937 | A | T | 0.034 | 6.24E-05 |
| rs35614134 | A | C | 0.039 | 2.30E-05 |
| rs663129 | A | G | 0.04 | 1.82E-05 |
| rs116843064 | G | A | 0.159 | 2.87E-07 |
| rs111397563 | T | C | 0.052 | 1.28E-08 |
| rs6511720^#^ | G | T | 0.128 | 7.88E-22 |
| rs2738448 | G | C | 0.034 | 5.04E-05 |
| rs167479 | G | T | 0.04 | 2.26E-06 |
| rs73015715 | T | C | 0.049 | 2.32E-06 |
| rs78030362 | G | A | 0.069 | 5.46E-05 |
| rs10423964 | T | C | 0.039 | 2.38E-05 |
| rs10417115 | C | T | 0.068 | 2.25E-05 |
| rs34322801 | C | G | 0.05 | 6.03E-06 |
| rs73045269 | T | C | 0.064 | 1.71E-07 |
| rs4760 | G | A | 0.054 | 2.56E-05 |
| rs7412^#^ | C | T | 0.143 | 2.17E-19 |
| rs56131196 | A | G | 0.082 | 2.71E-12 |
| rs1964272 | G | A | 0.044 | 2.29E-07 |
| rs425105 | C | T | 0.047 | 4.72E-05 |
| rs13734 | A | G | 0.043 | 2.03E-05 |
| rs59909520 | C | T | 0.059 | 1.04E-05 |
| rs867186 | A | G | 0.057 | 1.47E-05 |
| rs117113213 | A | G | 0.131 | 1.19E-06 |
| rs6129767 | G | T | 0.04 | 1.04E-05 |
| rs56313611 | C | T | 0.058 | 1.39E-06 |
| rs259983 | C | A | 0.056 | 2.89E-06 |
| rs3813452 | T | C | 0.035 | 3.67E-05 |
| rs2832275 | T | A | 0.051 | 2.04E-06 |
| rs75187018 | G | A | 0.136 | 1.85E-05 |
| rs28451064^#^ | A | G | 0.133 | 2.62E-23 |
| rs743339 | C | T | 0.075 | 3.05E-14 |
| rs117696200 | T | G | 0.079 | 1.82E-05 |
| rs2836621 | T | C | 0.033 | 4.68E-05 |
| rs35219138 | C | A | 0.034 | 4.83E-05 |
| rs9604969 | A | G | 0.06 | 5.69E-05 |
| rs71313931 | G | C | 0.039 | 1.87E-05 |
| rs11287675 | C | T | 0.035 | 2.78E-05 |
| rs12485143 | C | T | 0.067 | 2.72E-05 |
| rs468224 | A | G | 0.043 | 8.85E-06 |

Note: Effect estimates and p-values reported here were obtained from previous genome-wide association studies.^4, 21^ “#” indicates 46 SNPs which were genome-wide significant at a p-value of 5×10^-8^ and in low linkage disequilibrium defined according to r^2^<0.001.

Supplemental Table 2. Associations of TV viewing and computer use with incident coronary heart disease (CHD) after excluding an additional two years of follow-up.

| Comparisons | Number of participants | Number of cases | Crude incident rate per 100,000-person years | Hazard ratio  (95% confidence interval) |
| --- | --- | --- | --- | --- |
|  | 371,705 | 7,864 | 167.3 |  |
| **Categories of TV viewing** |  |  |  |  |
| ≥4hours/day (Reference) | 105,972 | 2,963 | 221.9 | 1.00 (Reference) |
| 2-3hours/day | 190,936 | 3,794 | 157.0 | 0.96 (0.91-1.01) |
| 0-1hour/day | 74,797 | 1,107 | 116.7 | 0.87 (0.81-0.94) |
|  |  |  |  |  |
| **Categories of computer use** |  |  |  |  |
| ≥4hours/day (Reference) | 19,176 | 438 | 181.9 | 1.00 (Reference) |
| 2-3hours/day | 60,339 | 1,462 | 193.2 | 0.99 (0.89-1.10) |
| 0-1hour/day | 292,190 | 5,964 | 161.1 | 0.98 (0.89-1.08) |

Models were adjusted for sex, body mass index (weight in kilograms/height in meters squared), smoking status (never, previous, current), employment (unemployed, employed), Townsend Deprivation Index (a numerical deprivation score generated based on employment, car ownership, home ownership and household overcrowding according to postcode of participants’ home address), alcohol consumption (never, previous, currently <3 times/week, currently ≥3 times/week), salt-adding behaviour (never/rarely, sometimes, usually, always), oily fish consumption (never, <once/week, once/week, >once/week), coffee intake (cups/day), fruit and vegetable intake (a composite score generated based on intake of fresh/dried fruit and intake of raw/cooked vegetable ranging from 0 to 4), processed/red meat intake (days/week), hypertension medication use, cholesterol-lowering medication use, glucose-lowering medication use, sleep (≤5, 6, 7, 8 and ≥9hours/day) and moderate-to-vigorous physical activity (minutes/day): plus the polygenic risk score, genotype array type and first ten principal components of genetic ancestry

Supplemental Table 3. Associations of TV viewing and computer use with incident coronary heart disease (CHD) after excluding individuals with poor self-reported health status (i.e. based on the 4-level self-reported health ratings; poor [excluded], fair, good, excellent)

| Comparisons | Number of participants | Number of cases | Crude incident rate per 100,000-person years | Hazard ratio  (95% confidence interval) |
| --- | --- | --- | --- | --- |
|  | 359,691 | 8,433 | 185.8 |  |
| **Categories of TV viewing** |  |  |  |  |
| ≥4hours/day (Reference) | 99,877 | 3,061 | 243.9 | 1.00 (Reference) |
| 2-3hours/day | 186,462 | 4,166 | 176.9 | 0.95 (0.91-0.99) |
| 0-1hour/day | 73,352 | 1,206 | 129.8 | 0.85 (0.79-0.91) |
|  |  |  |  |  |
| **Categories of computer use** |  |  |  |  |
| ≥4hours/day (Reference) | 18,136 | 451 | 198.4 | 1.00 (Reference) |
| 2-3hours/day | 58,040 | 1,566 | 215.7 | 1.00 (0.91-1.10) |
| 0-1hour/day | 283,515 | 6,416 | 178.9 | 1.00 (0.91-1.10) |

Models were adjusted for sex, body mass index (weight in kilograms/height in meters squared), smoking status (never, previous, current), employment (unemployed, employed), Townsend Deprivation Index (a numerical deprivation score generated based on employment, car ownership, home ownership and household overcrowding according to postcode of participants’ home address), alcohol consumption (never, previous, currently <3 times/week, currently ≥3 times/week), salt-adding behaviour (never/rarely, sometimes, usually, always), oily fish consumption (never, <once/week, once/week, >once/week), coffee intake (cups/day), fruit and vegetable intake (a composite score generated based on intake of fresh/dried fruit and intake of raw/cooked vegetable ranging from 0 to 4), processed/red meat intake (days/week), hypertension medication use, cholesterol-lowering medication use, glucose-lowering medication use, sleep (≤5, 6, 7, 8 and ≥9hours/day) and moderate-to-vigorous physical activity (minutes/day): plus the polygenic risk score, genotype array type and first ten principal components of genetic ancestry

Supplemental Table 4. Associations of TV viewing and computer use with incident coronary heart disease (CHD) after excluding individuals with 2nd-degree genetic relatedness.

| Comparisons | Number of participants | Number of cases | Crude incident rate per 100,000-person years | Hazard ratio  (95% confidence interval) |
| --- | --- | --- | --- | --- |
|  | 316,054 | 7,745 | 194.5 |  |
| **Categories of TV viewing** |  |  |  |  |
| ≥4hours/day (Reference) | 89,165 | 2,899 | 259.2 | 1.00 (Reference) |
| 2-3hours/day | 163,592 | 3,763 | 183.5 | 0.95 (0.91-1.00) |
| 0-1hour/day | 64,297 | 1,083 | 133.1 | 0.84 (0.78-0.91) |
|  |  |  |  |  |
| **Categories of computer use** |  |  |  |  |
| ≥4hours/day (Reference) | 16,676 | 451 | 216.1 | 1.00 (Reference) |
| 2-3hours/day | 52,161 | 1,492 | 229.0 | 1.03 (0.93-1.15) |
| 0-1hour/day | 247,217 | 5,802 | 185.8 | 1.04 (0.94-1.14) |

Models were adjusted for sex, body mass index (weight in kilograms/height in meters squared), smoking status (never, previous, current), employment (unemployed, employed), Townsend Deprivation Index (a numerical deprivation score generated based on employment, car ownership, home ownership and household overcrowding according to postcode of participants’ home address), alcohol consumption (never, previous, currently <3 times/week, currently ≥3 times/week), salt-adding behaviour (never/rarely, sometimes, usually, always), oily fish consumption (never, <once/week, once/week, >once/week), coffee intake (cups/day), fruit and vegetable intake (a composite score generated based on intake of fresh/dried fruit and intake of raw/cooked vegetable ranging from 0 to 4), processed/red meat intake (days/week), hypertension medication use, cholesterol-lowering medication use, glucose-lowering medication use, sleep (≤5, 6, 7, 8 and ≥9hours/day) and moderate-to-vigorous physical activity (minutes/day): plus the polygenic risk score, genotype array type and first ten principal components of genetic ancestry

Supplemental Table 5. Associations of genetic risk for coronary heart disease and TV viewing and computer use with incident coronary heart disease (CHD) using a weighted polygenic risk score calculated based only on 46 lead SNPs (from 46 loci) which were genome-wide significant at a p-value of 5×10^-8^ and in low linkage disequilibrium defined according to r^2^<0.001

| Comparisons | Number of participants | Number of cases | Crude incident rate per 100,000-person years | Hazard ratio  (95% confidence interval) |
| --- | --- | --- | --- | --- |
|  | 373,026 | 9,185 | 195.3 |  |
| **Categories of TV viewing** |  |  |  |  |
| ≥4hours/day (Reference) | 106,501 | 3,501 | 261.8 | 1.00 (Reference) |
| 2-3hours/day | 191,555 | 4,413 | 182.5 | 0.94 (0.90-0.99) |
| 0-1hour/day | 74,961 | 1,271 | 133.9 | 0.84 (0.78-0.90) |
|  |  |  |  |  |
| **Categories of computer use** |  |  |  |  |
| ≥4hours/day (Reference) | 19,253 | 515 | 213.6 | 1.00 (Reference) |
| 2-3hours/day | 60,596 | 1,719 | 226.9 | 1.00 (0.91-1.10) |
| 0-1hour/day | 293,177 | 6,951 | 187.6 | 0.99 (0.91-1.09) |
|  |  |  |  |  |
| **Tertiles of genetic risk** |  |  |  |  |
| Low (Reference) | 124,342 | 2,329 | 148.2 | 1.00 (Reference) |
| Middle | 124,342 | 2,920 | 186.1 | 1.26 (1.20-1.33) |
| High | 124,342 | 3,936 | 251.7 | 1.72 (1.63-1.81) |

Models were adjusted for sex, body mass index (weight in kilograms/height in meters squared), smoking status (never, previous, current), employment (unemployed, employed), Townsend Deprivation Index (a numerical deprivation score generated based on employment, car ownership, home ownership and household overcrowding according to postcode of participants’ home address), alcohol consumption (never, previous, currently <3 times/week, currently ≥3 times/week), salt-adding behaviour (never/rarely, sometimes, usually, always), oily fish consumption (never, <once/week, once/week, >once/week), coffee intake (cups/day), fruit and vegetable intake (a composite score generated based on intake of fresh/dried fruit and intake of raw/cooked vegetable ranging from 0 to 4), processed/red meat intake (days/week), hypertension medication use, cholesterol-lowering medication use, glucose-lowering medication use, sleep (≤5, 6, 7, 8 and ≥9hours/day), moderate-to-vigorous physical activity (minutes/day), the polygenic risk score, genotype array type and first ten principal components of genetic ancestry, with mutual adjustment of TV viewing and computer use in models using either TV viewing or computer use as the main exposure; and adjusted for sex, genotype array type and first ten principal components of genetic ancestry in models using polygenic risk scores as the main exposure.

Supplemental Table 6. Associations of TV viewing and computer use with incident coronary heart disease (CHD) using values imputed for the covariates missing, assuming data missing at random

| Comparisons | Number of participants | Number of cases | Crude incident rate per 100,000-person years | Hazard ratio  (95% confidence interval) |
| --- | --- | --- | --- | --- |
|  | 389,234 | 9,765 | 199.0 |  |
| **Categories of TV viewing** |  |  |  |  |
| ≥4hours/day (Reference) | 113,575 | 3,802 | 266.7 | 1.00 (Reference) |
| 2-3hours/day | 198,546 | 4,635 | 189.0 | 0.94 (0.90-0.99) |
| 0-1hour/day | 77,113 | 1,328 | 136.0 | 0.85 (0.79-0.90) |
|  |  |  |  |  |
| **Categories of computer use** |  |  |  |  |
| ≥4hours/day (Reference) | 19,953 | 542 | 216.9 | 1.00 (Reference) |
| 2-3hours/day | 63,018 | 1,807 | 229.4 | 1.00 (0.91-1.10) |
| 0-1hour/day | 306,263 | 7,416 | 191.6 | 0.99 (0.91-1.08) |

Models were adjusted for sex, body mass index (weight in kilograms/height in meters squared), smoking status (never, previous, current), employment (unemployed, employed), Townsend Deprivation Index (a numerical deprivation score generated based on employment, car ownership, home ownership and household overcrowding according to postcode of participants’ home address), alcohol consumption (never, previous, currently <3 times/week, currently ≥3 times/week), salt-adding behaviour (never/rarely, sometimes, usually, always), oily fish consumption (never, <once/week, once/week, >once/week), coffee intake (cups/day), fruit and vegetable intake (a composite score generated based on intake of fresh/dried fruit and intake of raw/cooked vegetable ranging from 0 to 4), processed/red meat intake (days/week), hypertension medication use, cholesterol-lowering medication use, glucose-lowering medication use, sleep (≤5, 6, 7, 8 and ≥9hours/day) and moderate-to-vigorous physical activity (minutes/day): plus the polygenic risk score, genotype array type and first ten principal components of genetic ancestry

Supplemental Table 7. Associations of TV viewing and computer use with incident coronary heart disease (CHD) including prevalence of type 2 diabetes and renal dysfunction as potential confounders

| Comparisons | Number of participants | Number of cases | Crude incident rate per 100,000-person years | Hazard ratio  (95% confidence interval) |
| --- | --- | --- | --- | --- |
|  | 372,402 | 9,163 | 195.1 |  |
| **Categories of TV viewing** |  |  |  |  |
| ≥4hours/day (Reference) | 106,236 | 3,487 | 261.5 | 1.00 (Reference) |
| 2-3hours/day | 191,285 | 4,407 | 182.5 | 0.94 (0.90-0.99) |
| 0-1hour/day | 75,881 | 1,269 | 133.8 | 0.84 (0.79-0.90) |
|  |  |  |  |  |
| **Categories of computer use** |  |  |  |  |
| ≥4hours/day (Reference) | 19,200 | 513 | 213.4 | 1.00 (Reference) |
| 2-3hours/day | 60,494 | 1,715 | 226.8 | 1.01 (0.91-1.11) |
| 0-1hour/day | 292,708 | 6,935 | 187.5 | 1.00 (0.91-1.09) |

Models were adjusted for sex, body mass index (weight in kilograms/height in meters squared), smoking status (never, previous, current), employment (unemployed, employed), Townsend Deprivation Index (a numerical deprivation score generated based on employment, car ownership, home ownership and household overcrowding according to postcode of participants’ home address), alcohol consumption (never, previous, currently <3 times/week, currently ≥3 times/week), salt-adding behaviour (never/rarely, sometimes, usually, always), oily fish consumption (never, <once/week, once/week, >once/week), coffee intake (cups/day), fruit and vegetable intake (a composite score generated based on intake of fresh/dried fruit and intake of raw/cooked vegetable ranging from 0 to 4), processed/red meat intake (days/week), hypertension medication use, cholesterol-lowering medication use, glucose-lowering medication use, sleep (≤5, 6, 7, 8 and ≥9hours/day), moderate-to-vigorous physical activity (minutes/day), type 2 diabetes (yes/no) and renal dysfunction (yes/no): plus the polygenic risk score, genotype array type and first ten principal components of genetic ancestry

Supplemental Table 8. Associations of TV viewing and computer use with incident coronary heart disease (CHD) excluding body mass index as a potential confounder

| Comparisons | Number of participants | Number of cases | Crude incident rate per 100,000-person years | Hazard ratio  (95% confidence interval) |
| --- | --- | --- | --- | --- |
|  | 374,019 | 9,213 | 195.3 |  |
| **Categories of TV viewing** |  |  |  |  |
| ≥4hours/day (Reference) | 106,909 | 3,520 | 262.3 | 1.00 (Reference) |
| 2-3hours/day | 191,973 | 4,421 | 182.5 | 0.91 (0.87-0.96) |
| 0-1hour/day | 75,137 | 1,272 | 133.7 | 0.79 (0.74-0.85) |
|  |  |  |  |  |
| **Categories of computer use** |  |  |  |  |
| ≥4hours/day (Reference) | 19,342 | 517 | 213.5 | 1.00 (Reference) |
| 2-3hours/day | 60,814 | 1,729 | 227.5 | 0.99 (0.90-1.09) |
| 0-1hour/day | 293,863 | 6,967 | 187.6 | 0.96 (0.88-1.05) |

Models were adjusted for sex, smoking status (never, previous, current), employment (unemployed, employed), Townsend Deprivation Index (a numerical deprivation score generated based on employment, car ownership, home ownership and household overcrowding according to postcode of participants’ home address), alcohol consumption (never, previous, currently <3 times/week, currently ≥3 times/week), salt-adding behaviour (never/rarely, sometimes, usually, always), oily fish consumption (never, <once/week, once/week, >once/week), coffee intake (cups/day), fruit and vegetable intake (a composite score generated based on intake of fresh/dried fruit and intake of raw/cooked vegetable ranging from 0 to 4), processed/red meat intake (days/week), hypertension medication use, cholesterol-lowering medication use, glucose-lowering medication use, sleep (≤5, 6, 7, 8 and ≥9hours/day) and moderate-to-vigorous physical activity (minutes/day): plus the polygenic risk score, genotype array type and first ten principal components of genetic ancestry

Supplemental Table 9. Associations of TV viewing and computer use with incident coronary heart disease (CHD) using CHD follow-up censored on January 1st, 2020 to account for potential CHD cases not captured due to participants’ fear of visiting clinics during COVID-19.

| Comparisons | Number of participants | Number of cases | Crude incident rate per 100,000-person years | Hazard ratio  (95% confidence interval) |
| --- | --- | --- | --- | --- |
|  | 373,026 | 7,880 | 194.5 |  |
| **Categories of TV viewing** |  |  |  |  |
| ≥4hours/day (Reference) | 106,510 | 3,042 | 263.9 | 1.00 (Reference) |
| 2-3hours/day | 191,555 | 3,756 | 180.4 | 0.92 (0.88-0.97) |
| 0-1hour/day | 74,961 | 1,082 | 132.4 | 0.84 (0.78-0.91) |
|  |  |  |  |  |
| **Categories of computer use** |  |  |  |  |
| ≥4hours/day (Reference) | 19,253 | 432 | 208.2 | 1.00 (Reference) |
| 2-3hours/day | 60,596 | 1,478 | 226.7 | 1.02 (0.92-1.14) |
| 0-1hour/day | 294,177 | 5,970 | 187.0 | 1.01 (0.92-1.12) |

Models were adjusted for sex, body mass index (weight in kilograms/height in meters squared), smoking status (never, previous, current), employment (unemployed, employed), Townsend Deprivation Index (a numerical deprivation score generated based on employment, car ownership, home ownership and household overcrowding according to postcode of participants’ home address), alcohol consumption (never, previous, currently <3 times/week, currently ≥3 times/week), salt-adding behaviour (never/rarely, sometimes, usually, always), oily fish consumption (never, <once/week, once/week, >once/week), coffee intake (cups/day), fruit and vegetable intake (a composite score generated based on intake of fresh/dried fruit and intake of raw/cooked vegetable ranging from 0 to 4), processed/red meat intake (days/week), hypertension medication use, cholesterol-lowering medication use, glucose-lowering medication use, sleep (≤5, 6, 7, 8 and ≥9hours/day) and moderate-to-vigorous physical activity (minutes/day): plus the polygenic risk score, genotype array type and first ten principal components of genetic ancestry

Supplemental Table 10. Associations of TV viewing and computer use with incident coronary heart disease (CHD) with adjustment for education, household income and occupation as individual-level indicators of socio-economic status as opposed to area-level socio-economic status (Townsend Deprivation Index).

| Comparisons | Number of participants | Number of cases | Crude incident rate per 100,000-person years | Hazard ratio  (95% confidence interval) |
| --- | --- | --- | --- | --- |
|  | 278,742 | 5,976 | 169.9 |  |
| **Categories of TV viewing** |  |  |  |  |
| ≥4hours/day (Reference) | 65,141 | 1,906 | 233.2 | 1.00 (Reference) |
| 2-3hours/day | 149,120 | 3,063 | 162.7 | 0.93 (0.88-0.99) |
| 0-1hour/day | 64,481 | 1,007 | 123.3 | 0.87 (0.80-0.94) |
|  |  |  |  |  |
| **Categories of computer use** |  |  |  |  |
| ≥4hours/day (Reference) | 16,065 | 403 | 200.2 | 1.00 (Reference) |
| 2-3hours/day | 48,784 | 1,290 | 211.5 | 1.00 (0.89-1.11) |
| 0-1hour/day | 213,893 | 4,283 | 158.3 | 0.95 (0.86-1.06) |

Models were adjusted for sex, body mass index (weight in kilograms/height in meters squared), smoking status (never, previous, current), employment (unemployed, employed), education (College or University degree, A levels/AS levels or equivalent, O level/GCSEs or equivalent, CSEs or equivalent, NVQ or HND or HNC or equivalent, Other professional qualifications), household income (less than £18,000£, £18,000 to £30,999, £31,000 to £51,999, £52,000 to £100,000, greater than £100,000) and occupation (in paid employment or self-employed, retired, looking after home and/or family, unable to work because of sickness or disability, unemployed, doing unpaid or voluntary work, full or part-time student), alcohol consumption (never, previous, currently <3 times/week, currently ≥3 times/week), salt-adding behaviour (never/rarely, sometimes, usually, always), oily fish consumption (never, <once/week, once/week, >once/week), coffee intake (cups/day), fruit and vegetable intake (a composite score generated based on intake of fresh/dried fruit and intake of raw/cooked vegetable ranging from 0 to 4), processed/red meat intake (days/week), hypertension medication use, cholesterol-lowering medication use, glucose-lowering medication use, sleep (≤5, 6, 7, 8 and ≥9hours/day) and moderate-to-vigorous physical activity (minutes/day): plus the polygenic risk score, genotype array type and first ten principal components of genetic ancestry
